# Molecular Modeling Studies on 11*H*-Dibenz[*b*,*e*]azepine and Dibenz[*b*,*f*][1,4]oxazepine Derivatives as Potent Agonists of the Human TRPA1 Receptor

**DOI:** 10.3390/molecules15129364

**Published:** 2010-12-17

**Authors:** Yong Ai, Fa-Jun Song, Shao-Teng Wang, Qiang Sun, Ping-Hua Sun

**Affiliations:** 1 College of Pharmacy, South Central University for Nationalities, 708 Minyuan Road, Wuhan 430074, China; E-Mails: aiyong0508@126.com (Y.A.); wst418638862@sohu.com (S.-T.W.); sqiang6196@126.com (Q.S.); 2 College of Life Sciences, South Central University for Nationalities, 708 Minyuan Road, Wuhan 430074, China; 3 Guangdong Province Key Laboratory of Pharmacodynamic Constituents of TCM and New Drugs Research, College of Pharmacy, Jinan University, Guangzhou 510632, China; E-Mail: Pinghuasunny@163.com (P-H.S.)

**Keywords:** 11*H*-dibenz[*b*,*e*]azepines, dibenz[*b*,*f*][1,4]oxazepines, CoMFA, CoMSIA, TRPA1

## Abstract

A computational strategy based on comparative molecular fields analysis (CoMFA) and comparative molecular similarity indices analysis (CoMSIA) was performed on a series of the 11*H*-dibenz[*b*,*e*]azepine and dibenz[*b*,*f*][1,4]oxazepine derivatives as potent agonists of the human TRPA1 receptor. The CoMFA and CoMSIA models resulting from a 21 molecule training set gave *r*^2^_cv_ values of 0.631 and 0.542 and *r^2^* values of 0.986 and 0.981, respectively. The statistically significant models were validated by a test set of five compounds with predictive *r*^2^_pred_. values of 0.967 and 0.981 for CoMFA and CoMSIA, respectively. A systemic external validation was also performed on the established models. The information obtained from 3D counter maps could facilitate the design of more potent human TRPA1 receptor agonists.

## 1. Introduction

The transient receptor potential ankyrin 1 (TRPA1) receptor is a member of the transient receptor potential (TRP) family of the cation-selection channel and the only mammalian member of the TRPA subfamily [1,2]. It is expressed in the dorsal root ganglion, trigeminal ganglion (TG) neurons [3], and non-sensory tissue [4]. It plays an essential role as a biological sensor to irritant chemicals [5] and is implicated in a growing number of diseases, such as bladder disorders [6], inflammatory pain [7], and airway diseases [8]. The activation of TRPA1 by a diversity of chemical agents is widely accepted. It can be activated by many pungent chemicals, including methyl salicylate (from wintergreen oil) [3], isothiocyanates like allylisothiocyanate, the pungent compound in mustard oil (MO), wasabi, and horseradish [3], cinnamaldehyde [3], Δ9-tetrahydrocannabinol (Δ9-THC, the psychoactive compound in marijuana) [9], allicin and diallyl disulphide (from garlic) [10], acrolein (an irritant found in vehicle exhaust fumes and tear gas) [10], and the lacrimators 1-chloroacetophenone (CN), dibenz[*b*,*f*]- [1,4]oxazepine (CR) and 2-chlorobenzylidene malononitrile (CS) [11]. Most of the known activating compounds contain reactive, electrophilic chemical groups that react with cysteine residues in the active site of the TRPA1 channel [11]. A considerable amount of investigation has been carried out to develop reversible ligands that target TRPA1 receptor and several agonists have been under pharmacological evaluation. Recently, a series of compounds containing 11*H*-dibenz[*b*,*e*]azepines, and dibenz[*b*,*f* ][1,4]oxazepines that function as extremely potent activators of the human TRPA1 receptor activities were reported by literature [5].

In this paper, molecular modeling studies of these 11*H*-dibenz[*b*,*e*]azepine and dibenz[*b*,*f*][1,4]- oxazepine derivatives were performed using 3D-QSAR approaches. Thus, 3D-QSAR, including comparative molecular field analysis (CoMFA) and comparative molecular similarity indices analysis (CoMSIA) methods, were performed to predict the agonistic activities of these agonists and provide the regions in space where interactive fields may influence the activity. Furthermore, the 3D-QSAR models were validated by external validation. The constructed models can not only help in understanding the structure-activity relationship of these compounds but also may serve as a useful guide for the design of new agonists with much higher agonistic potencies. 

## 2. Results and Discussion

A data set of 21 diverse analogues was selected as a training set to derive the conventional CoMFA and CoMSIA models and an additional five compounds (indicated with “*” in Table 2) were used to test the accuracy of these models. Selection of the training set and test set molecules was done randomly. The structures and associated biological activities are shown in Table 1 and Table 2.

### 2.1. CoMFA PLS Analysis

The CoMFA model of 11*H*-dibenz[*b*,*e*]azepines and dibenz[*b*,*f* ][1,4]oxazepines derivatives gave a cross-validated correlation coefficient (*r^2^_cv_*) of 0.631 (>0.5) with an optimized component of 6, which indicated that this model may be considerably reliable to predict the pEC_50_ values of the compouds of the test set.

**Table 1:** The structures of the training and test set molecules. 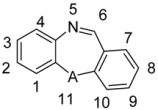

Compd.No.	Substituent	
	R	A
**1**	H	O
**2**	H	CH_2_
**3**	1-COOMe	CH_2_
**4**	2-COOMe	CH_2_
**5**	3-COOMe	CH_2_
**6**	4-COOMe	CH_2_
**7**	7-COOMe	CH_2_
**8**	8-COOMe_2_	CH_2_
**9**	9-COOMe	O
**10**	10-COOMe	O
**11**	8-Br	CH_2_
**12**	10-Br	CH_2_
**13**	8-CN	CH_2_
**14**	10-CN	CH_2_
**15**	10-CN	O
**16**	1-CN	O
**17**	10-CONH_2_	CH_2_
**18**	10-CONH_2_	O
**19**	1-CONH_2_	O
**20**	8-COO *^i^*Pr	CH_2_
**21**	10-COOnBu	CH_2_
**22**	10-CONH(CH_2_)_3_OMe	CH_2_
**23**	10-CONEt_2_	CH_2_
**24**	9-OMe	O
**25**	9-OH	O
**26**	H	S

**Table 2:** The actual pEC_50_s, predicted pEC_50_s (Pred.) and their residuals (Res.) of the training and test set molecules.

Compd.No.	pEC_50_	CoMFA		CoMSIA	
	Actual	Pred.	Res.	Pred.	Res.
**1**	9.523	9.548	−0.025	9.53	−0.007
**2**	8.523	8.111	0.412	7.991	0.532
**3**	9.222	9.191	0.031	9.279	−0.057
**4**	9.398	9.454	−0.056	9.317	0.081
**5**	7.721	7.683	0.038	7.82	−0.099
**6**	7.509	7.587	−0.078	7.526	−0.017
7*	7.509	7.688	−0.179	7.608	−0.099
**8**	7.000	6.982	0.018	7.028	−0.028
**9**	8.222	8.205	0.017	8.124	0.098
**10**	10.301	10.355	−0.054	10.248	0.053
**11**	7.482	7.532	−0.051	7.541	−0.060
**12**	8.456	8.433	0.023	8.438	0.018
13*	6.801	7.173	−0.372	7.102	−0.301
**14**	8.796	8.762	0.034	8.997	−0.201
15*	9.824	9.811	0.013	9.953	−0.129
16*	10.000	9.975	0.025	10.006	−0.006
**17**	8.602	8.774	−0.172	8.619	−0.017
**18**	10.097	9.989	0.108	10.045	0.052
**19**	9.959	9.92	0.039	9.939	0.020
**20**	6.030	6.054	−0.024	5.987	0.043
**21**	8.208	8.164	0.044	8.195	0.013
**22**	8.114	8.096	0.018	8.138	−0.025
**23**	7.398	7.401	−0.003	7.394	0.004
**24**	7.854	7.858	−0.004	7.995	−0.141
25*	8.678	8.9	−0.222	8.527	0.151
**26**	8.046	8.36	−0.314	8.306	−0.260

* Test set molecules.

Moreover, it gave a high non-cross-validated correlation coefficient (*r^2^*) of 0.986 with a low standard error estimate (SEE) of 0.154, and excellent *F* value of 160.559. Contributions of steric and electrostatic fields were 0.541 and 0,459, respectively. All statistical parameters associated with CoMFA model are listed in Table 3. 

**Figure 1:**
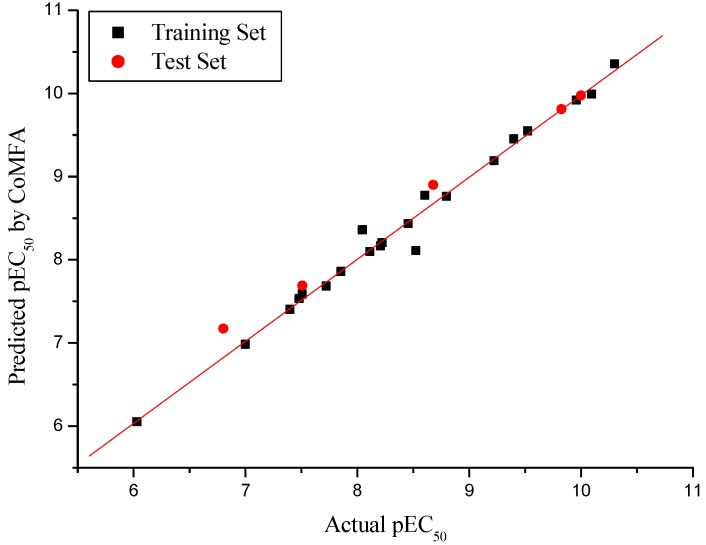
Graph of actual *versus* predicted pEC_50_ of the training set and the test set using CoMFA

The actual and predicted pEC_50_ values of the training set and test set by the model were given in Table 2, and the graph of actual activity *versus* predicted pEC_50_ of the training set and test set is illustrated in Figure 1.

**Table 3:** PLS results of CoMFA and CoMSIA models.

PLS Statistics	CoMFA	CoMSIA
*r*^2^_cv_^a^	0.631	0.542
*r*^2b^	0.986	0.981
ONC^c^	6	6
SEE^d^	0.154	0.180
F value^e^	160.559	117.466
Field contribution		
Steric	0.541	0.146
Electrostatic	0.459	0.403
Hydrophobic	-	0.160
H-bond Donor	-	0.075
H-bond Acceptor	-	0.215

^a^ cross-validated correlation coefficient; ^b^ non-cros-validated coefficient; ^c^ optimal number of components; ^d^ standard error of estimate; ^e^ value F-test value.

### 2.2. CoMSIA PLS Analysis

**Figure 2:**
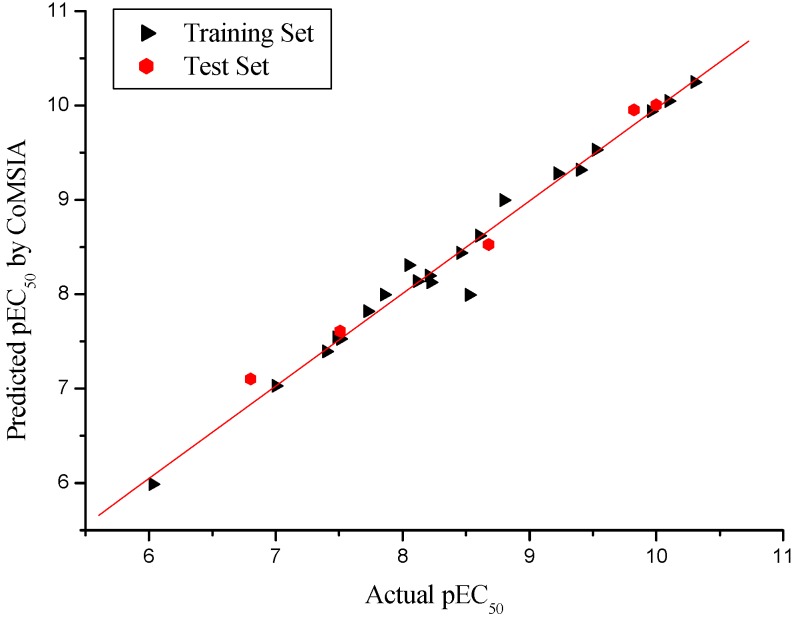
Graph of actual *versus* predicted pEC_50_ of the training set and the test set using CoMSIA.

The CoMSIA model consisting of steric (S), electrostatic (E), hydrophobic (H), hydrogen bond donor (D) and acceptor (A) fields gave a good leave-one-out correlation coefficient (*r^2^_cv_*) of 0.542 (>0.5) with an optimized component of 6, a high non-cross-validated correlation coefficient (*r^2^*) of 0.981 with a standard error estimate (SEE) of 0.180, and *F* value of 117.466. Contributions of steric, electrostatic, hydrophobic, hydrogen bond donor and hydrogen bond acceptor fields were 0.146, 0.403, 0.160, 0.075 and 0.215, respectively. All the statistical parameters supporting CoMSIA model are listed in Table 3. The actual and predicted pEC_50_ values and residual values for training set and test set compounds are given in Table 2. The relationship between actual and predicted pEC_50_ of the training set and test set compounds is illustrated in Figure 2.

### 2.3. External Validation Analysis for the CoMFA and CoMSIA

As can be seen from Table 4, the predictive correlation coefficient (*r*^2^_pred_) values based on molecules of the test set were 0.967 and 0.981 for the CoMFA and CoMSIA models, respectively. 

**Table 4:** Statistical parameters of the external validation.

Parameters (Testing set)	CoMFA	CoMSIA
*r*^2^_pred_	0.967	0.981
*Slope k*	0.984	0.988
*r*_0_^2^	0.984	0.988
*r*_m_^2^	0.942	0.899
[(*r*^2^-*r*_0_^2^)/*r*^2^]	0.002	-0.007

The correlation coefficients for regressions through the origin (actual and predicted activities), *r_0_^2^*, were 0.984 and 0.988, respectively. Excellent *r*_m_^2 ^values, 0.942 and 0.899 (>0.5) for the CoMFA and CoMSIA models, respectively, were obtained. The slopes of the regression lines of models *k* were 0.984 and 0.988 (0.85 ≤ k ≤ 1.15), which are close to 1. Moreover, the calculated [(*r*^2^ − *r*_0_^2^) / *r*^2^] values were 0.002 and −0.007 (<0.1) or the CoMFA and CoMSIA models, respectively. All the data discussed above suggested the fact that both CoMFA and CoMSIA models have not only good estimation abilities, but also robust predictive powers.

### 2.4. CoMFA Contour Maps Analysis

**Figure 3:**
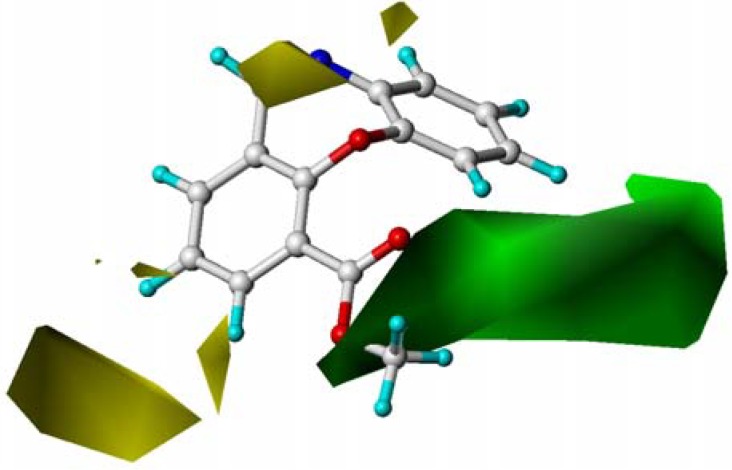
Contour maps of CoMFA steric regions in combination with compound **10**. Steric field: Green contours (80% contribution) indicate regions where bulky groups increase activity, while yellow contours (20% contribution) indicate regions where bulky groups decrease activity. Compound 10 is depicted in ball and stick representation, coloured by atom type (white C, blue N, red O, cyan H).

To visualize the information of the derived 3D-QSAR models, CoMFA contour maps were generated by plotting the coefficients from the CoMFA model. These contour maps may help identify important regions where changes in the steric and electrostatic fields were predicted to increase or decrease the activity. As shown in Figure 3, the yellow contour around the C-8 position signified that a bulky substituent at this site would decrease potency. This is consistent with the fact that the analogues modified at C-8 with bulky substituents such as compounds **8** (8-COOMe, pEC_50_ = 7.000), **11** (8-Br, pEC_50_ = 7.482), **13** (8-CN, pEC_50_ = 6.801) and **20** (8-COO*^i^*Pr, pEC_50_ = 6.030, 10,000-fold decrease in potency compared to compound 10) showed relatively decreased activities. The yellow contour near the C-9 indicated that a small group at this site would be favorable. 

This may also explain why derivative **25** (pEC_50_ = 8.678) bearing a hydroxyl group at this position displayed significantly improved agonistic activity compared to compounds **9** (pEC_50_ = 8.222) with a methoxycarbonyl and **24** (pEC_50_ = 7.854) with a methoxyl group. The sterically favorable green contour on C-10 position revealed that bulky groups in this region would benefit the agonistic activity. This can be verified by the fact that compounds **10**, **15**, and **18** have the activity order **10** (10-COOMe, 11-O, pEC_50_ = 10.301) > **18** (10-CONH_2_, 11-O, pEC_50_ = 10.097) > **15** (10-CN, 11-O, pEC_50_ = 9.824). However, by comparing all the C-10 modified compounds, it was found that larger substituents on the C-10 position such as those in compounds **12** (10-Br, pEC_50_ = 8.456), **14** (10-CN, pEC_50_ = 8.796), **17** (10-CONH, pEC_50_ = 8.602), **21** (10-COOnBu, pEC_50_ = 8.208), **22** (10-CONH(CH_2_)_3_OMe, pEC_50_ = 8.114), and **23** (10-CONEt_2_, pEC_50_ = 7.398) also resulted in decreased potencies when the 11-O was replaced by 11-CH_2_. In addition, two small yellow contours around the C-6 and C-4 respectively which were not observed in the CoMSIA steric field indicated that the small groups at these two sites may be beneficial for agonistic activity.

**Figure 4:**
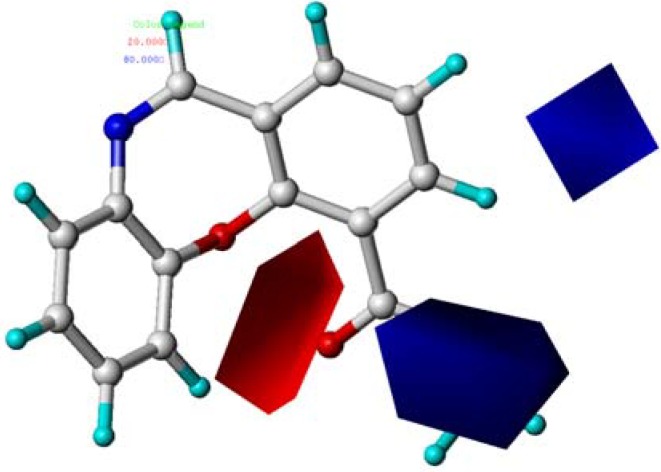
Contour maps of CoMFA electrostatic region in combination with compound **10**. Electrostatic fields: blue contours (80% contribution) represent regions where low electron density groups increase activity, while red contours (20% contribution) represent regions where high electron density groups increase activity. Compound **10** is depicted in ball and stick representation, coloured by atom type (white C, blue N, red O, cyan H).

As shown in Figure 4, the red contours around the 11 position (A) indicated that high electron density groups would be favorable. Hence, compounds **15** (10-CN, 11-O, pEC_50_ = 9.824) and **18** (10-CONH_2_, 11-O, pEC_50_ = 10.097), possessing O atom at 11 showed much better activity than those compounds with CH_2_ at 11 position (A) such as **14** (10-CN, 11-CH_2_, pEC_50 _= 8.796) and **17** (10-CONH_2_, 11-CH_2_, pEC_50_ = 8.602). The blue contour appeared at the middle of the C-8 position and C-9 position revealed that the low electron density group would be expected to benefit the potency. This was why compounds **08** (8-COOMe, pEC_50_ = 7.000), **09** (9-COOMe, pEC_50_ = 8.222), **11** (8-Br, pEC_50_ = 7.482), **13** (10-Br, pEC_50_ = 6.801), **20** (8-COO*^i^*Pr, pEC_50_ = 6.030), **24** (8-COOMe,pEC_50_ = 7.854), and **25** (9-OH, pEC_50 _= 8.678) with high electron density groups at C-8 or C-9 showed reduced agonistic potency.

### 2.5. CoMSIA Contour Maps

**Figure 5:**
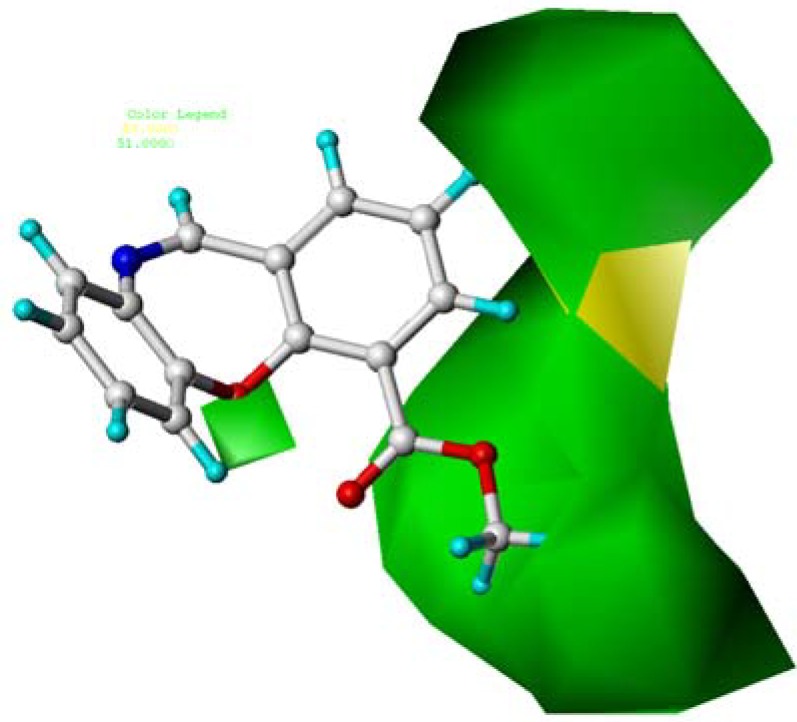
Contour maps of CoMSIA steric region in combination with compound **10**. Steric fields: green contours (51% contribution) indicate regions where bulky groups increase activity, while yellow contours (48% contribution) indicate regions where bulky groups decrease activity. Compound **10** is depicted in ball and stick representation, coloured by atom type (white C, blue N, red O, cyan H)

**Figure 6:**
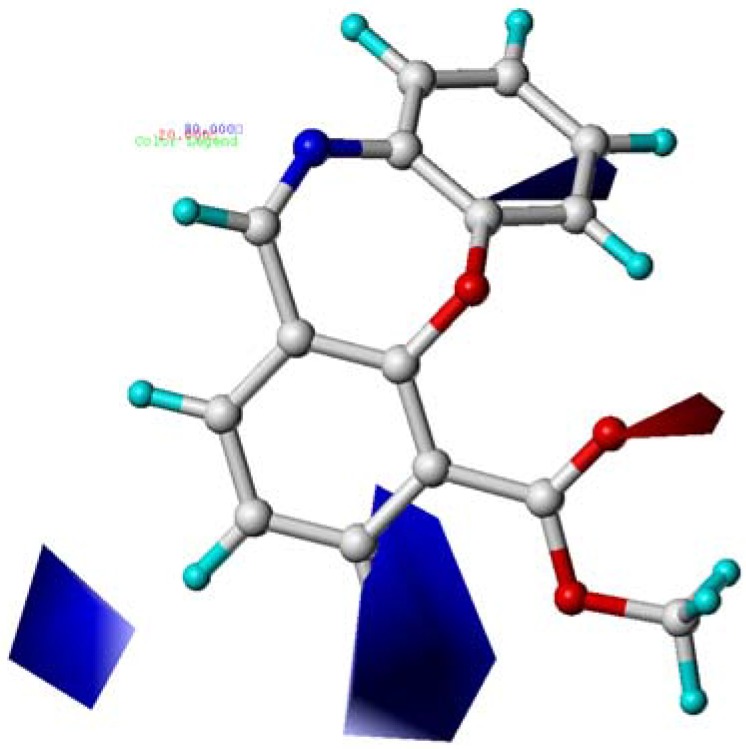
Contour maps of CoMSIA electrostatic region in combination with compound **10**. Electrostatic fields: blue contours (80% contribution) represent regions where low electron density groups increase activity, while red contours (20% contribution) represent regions where high electron density groups increase activity. Compound **10** is depicted in ball and stick representation, coloured by atom type (white C, blue N, red O, cyan H).

The steric (Figure 5) and electrostatic field (Figure 6) contour maps of the CoMSIA model were almost the same as the CoMFA-steric and electrostatic contours (Figure 3 and Figure 4). However, in the CoMFA model, two more contours appeared: one yellow contour map near the C-4 and a yellow contour map around the C-6 position.

**Figure 7:**
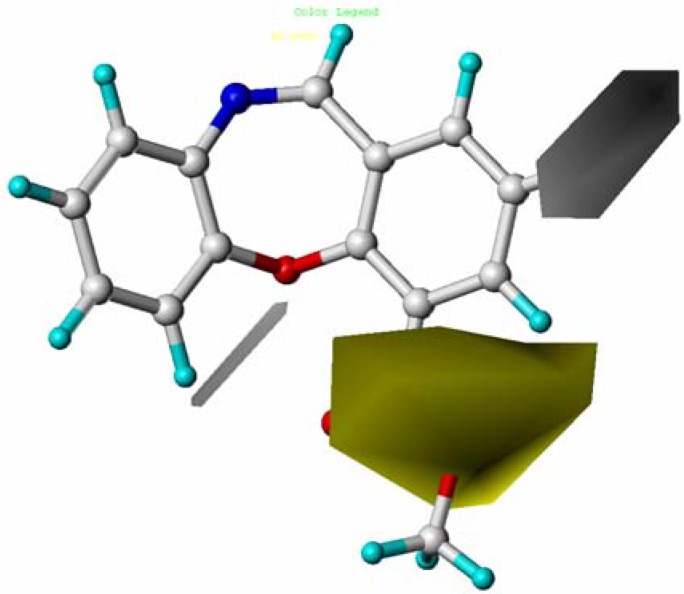
Contour maps of CoMSIA hydrophobic region in combination with compound **10**. Hydrophobic fields: The yellow and white (80% and 20% contributions) contours indicate favorable and unfavorable Hydrophobic groups. Compound **10** is depicted in ball and stick representation, coloured by atom type (white C, blue N, red O, cyan H)

The hydrophobic field is presented in Figure 7, yellow and white contours highlighted areas where hydrophobic and hydrophilic properties were preferred, respectively. The presence of a large yellow contour surrounding the 10-COOMe of the template molecule (compound **10**) indicated that hydro- phobic substituents may be well tolerated in that region.

The small white hydrophobic contour near the C-8 position indicated that hydrophobic groups were not suitable in this position. This may explain why compounds **8** (8-COOMe, pEC_50_ = 7.000) and **20** (8-COO*^i^*Pr, pEC_50_ = 6.030) showed significant decreased activities. Moreover, the white favored hydrophilic contour was observed on 11 position (A) where hydrophilic substituents were essential for activity. For example, the activity of the compounds **10** (pEC_50_ = 10.301), **15** (pEC_50_ = 9.824), **16** (pEC_50_ = 10.000), **18** (pEC_50_ = 10.097), **19** (pEC_50_ = 9.959), and **25** (pEC_50_ = 8.678) with O atoms displayed more potent EC_50_ values than compounds **8** (pEC_50_ = 7.000), **13** (pEC_50_ = 6.801),**20** (pEC_50_ = 6.030), and **23** (pEC_50_ = 7.398).

**Figure 8:**
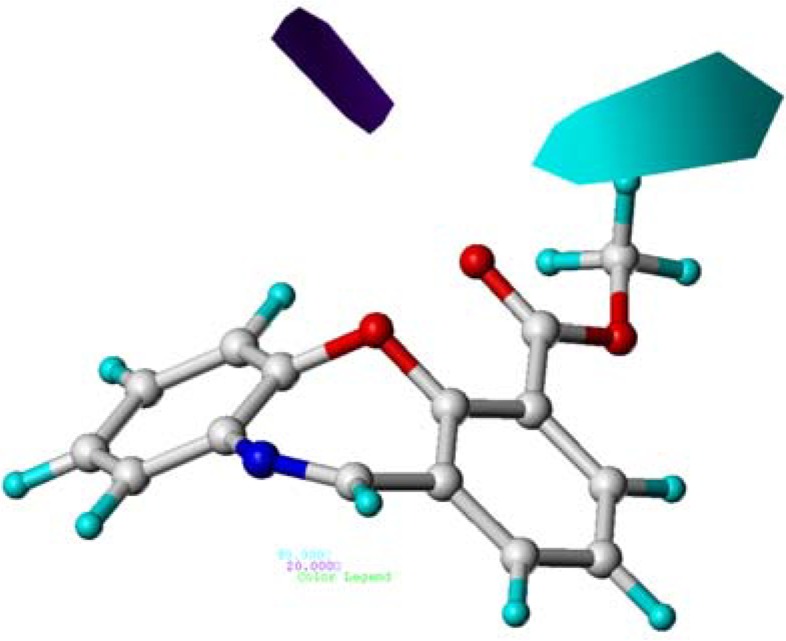
Contour maps of CoMSIA hydrogen bond donor region in combination with compound **10**. Hydrogen bond donor contour map. The cyan and purple (80% and 20% contributions) contours indicate favorable and unfavorable hydrogen bond donor groups. Compound **10** is depicted in ball and stick representation, coloured by atom type (white C, blue N, red O, cyan H).

The hydrogen bond donor field contour map of CoMSIA is shown in Figure 8 using compound **10** as a reference molecule. The cyan and purple contours represent favorable and unfavorable hydrogen bond donor groups.

In the CoMSIA hydrogen bond fields, the purple contour near the 11 position (A) and CO of the template molecule revealed that hydrogen bond donor groups may decrease the potency. In fact, the -O and -CO at this position acted as hydrogen bond acceptors, and this may explain why compounds **10** (10-COOMe, pEC_50_ = 10.301), and **18** (10-CONH_2_, pEC_50_ = 10.097) showed relative better activities. A hydrogen bond donor contours features, displayed by cyan contours near the terminal of 10-COOMe revealed that this group acted as a hydrogen bond donor and would be favored over the groups found in compounds such as **18** (10-CONH_2_, pEC_50_ = 10.097) and **19** (1-CONH_2_, pEC_50_ = 9.959).

**Figure 9:**
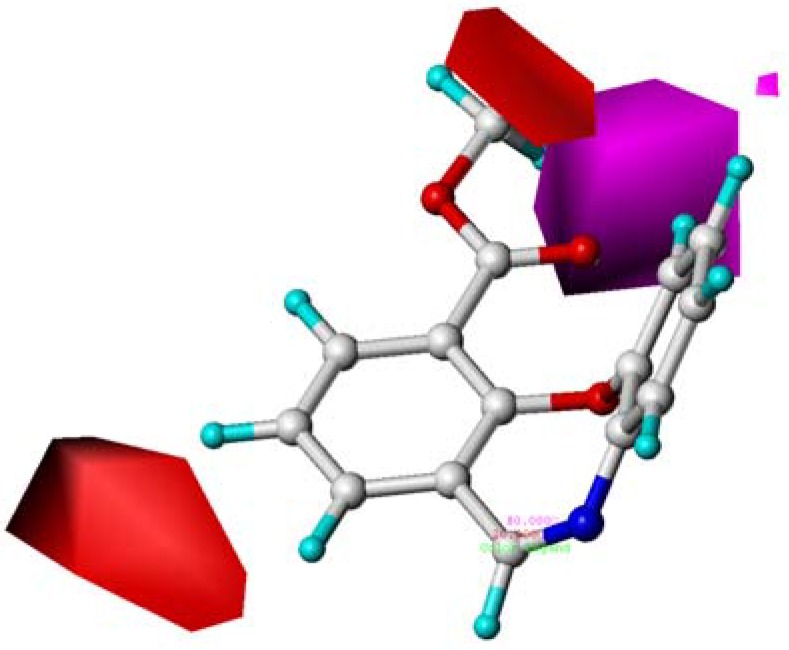
Contour maps of CoMSIA hydrogen bond acceptor region in combination with compound **10**. The magenta and red contours indicate favorable and unfavorable hydrogen bond acceptor groups. Compound **10** is depicted in ball and stick representation, coloured by atom type (white C, blue N, red O, cyan H).

The hydrogen bond acceptor field contour map of CoMSIA is shown in Figure 9 using compound **10** as a reference molecule. The magenta and red contours represent favorable and unfavorable hydrogen bond acceptor groups. In the CoMSIA hydrogen bond fields, the magenta contour near the 10-CO and 11-O revealed that hydrogen bond acceptor groups may benefit the potency. These results are supported by the evidence of the high potency of **10** (10-COOMe, pEC_50_ = 10.301), and **18** (10-CONH_2_, pEC_50_ = 10.097). A red contour around the terminal of 10-COOMe suggested that hydrogen bond acceptor groups may increase the agonistic activity. This may be the reason why compounds **18** (10-CONH_2_, pEC_50_ = 10.097) and **19** (1-CONH_2_, pEC_50_ = 9.959) showed relative increased activities.

### 2.6. Summary of the Structure-Activity Relationship Based on CoMFA and CoMSIA Models

**Figure 10:**
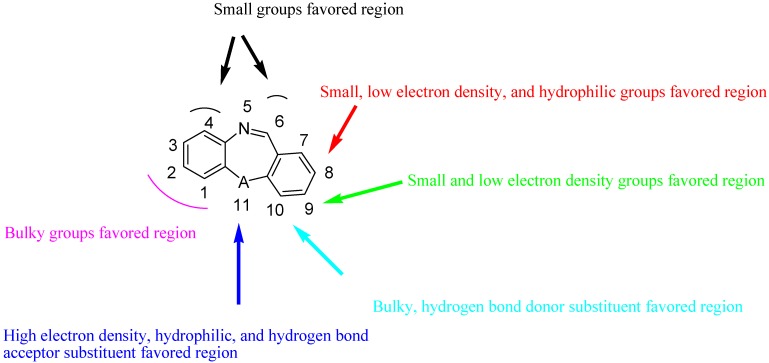
Structure-activity relationship revealed by 3D-QSAR studies.

The structure-activity relationships revealed by 3D-QSAR studies are illustrated in Figure 10. In detail, bulky groups at the C-1 and C-2 positions are favorable; small groups at C-4 and C-6 may be essential for the agonistic potency; small, low electron density, and hydrophilic groups at C-8 position could increase the agonistic activity; small and electron-donating substituents at C-9 may benefit the potency; bulky and hydrogen bond donor groups at C-10 would increase the potency; high electron density, hydrophilic, and hydrogen bond acceptor substituents at the 11 position (A) would be essential for the potency of the agonists.

## 3. Materials and Methods

### 3.1. Data Sets

The twenty-six compounds involved in this study were taken from the literature [5]. The associated activities were reported as EC_50 _values towards the human TRPA1 receptor. The EC_50 _values were converted into pEC_50 _by taking Log(1/EC_50_). From Table 2, the pEC_50_ values for the 26 studied agonists ranged from 6.030 to 10.301.

### 3.2. Molecular Modeling and Database Alignment

**Figure 11:**
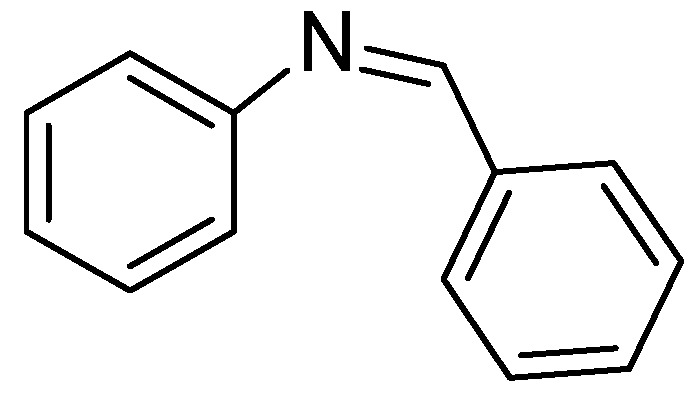
Common substructure used for alignment.

**Figure 12:**
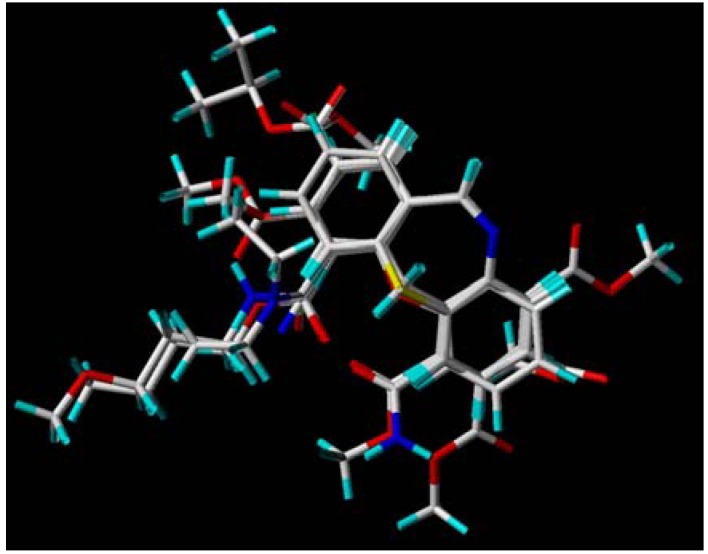
Alignment of the compounds used in the training set.

Molecular modeling and database alignment were performed by using the molecular modeling package SYBYL 8.1 (Tripos, Inc.) [12]. The 3D structures of all compounds were constructed by using the Sketch Molecule module. Energy minimization of each structure was performed using the SYBYL energy minimizer Tripos force field and Gasteiger-Hückel charge [13,14]. Molecular alignment was considered as one of the most sensitive parameters in 3D-QSAR analysis [15,16]. The quality and the predictive ability of the model are directly dependent on the alignment rule [17]. In this paper, all of the structures were aligned into a lattice box by fitting with (*Z*)-*N*-benzylidenebenzenamine group (Figure 11) as a common structure using compound **10** as a template, which was the most active compound. The aligned superimposed molecules are shown in Figure 12.

### 3.3. CoMFA Modeling

CoMFA is a widely used 3D-QSAR method which relates the biological activity of a series of molecules with their steric and electrostatic fields. The CoMFA descriptor fields were calculated at each lattice with grid spacing of 1 Å and extending to 4 Å units in all three dimensions within defined region [18,19]. The Van Der Waals potentials and Coulombic terms, which represented steric and electrostatic fields, respectively, were calculated by using the standard Tripos force field. In CoMFA method, a sp^3^ hybridized carbon atom with a charge of 1e was used as a probe atom, the energy values of the steric and electrostatic fields were truncated at 30 kcal/mol [19,20].

### 3.4. CoMSIA Modeling

The steric, electrostatic, hydrophobic, hydrogen bond donor and hydrogen bond acceptor CoMSIA potential fields were calculated at each lattice intersection of a regularly spaced grid of 1 Å and extending to 4 Å using a probe atom with radius 1.0 Å, +1.0 charge, and hydrophobic and hydrogen bond properties of +1. The attenuation factor was set to the default value of 0.3 [21,22].

### 3.5. PLS Analysis

The partial least-squares (PLS) approach, an extension of multiple regression analysis, was applied to linearly correlate the CoMFA and CoMSIA fields to the pEC_50_ values. CoMFA and CoMSIA descriptors were used as the independent variables. Column filtering was used at the default value of 2.0 kcal/mol in the cross-validation part.

The cross-validation analysis was performed using the leave-one-out (LOO) method in which one molecule was omitted from the dataset. The activity of the omitted molecule was then predicted by using the model derived from the rest of the dataset [23]. The leave-one-out (LOO) cross-validation method could check the predictivity of the obtained model and identify the optimum number of components (ONC). Thus the optimum number of components (ONC) was the number of components lead to the highest cross-validated correlated correlation coefficient *r*^2^ (*r*^2^_cv_) [23]. Finally, the CoMFA and CoMSIA models were generated using non-cross-validated PLS analysis with the optimum number of components (ONC) determined by the cross-validation. 

### 3.6. Models Validation-Predictive Correlation Co-Efficient (r^2^_pred_)

The predictive abilities of 3D-QSAR models were validated by predicting the activities of a test set of five compounds which were not included in the training set. These molecules were aligned to the template and their pEC_50_ values were predicted by the produced models which were obtained using the training set. The predictive correlation coefficient (*r*^2^_pred_), based on the molecules of test set, was calculated using the following Eq.:

*r*^2^_pred_ = (SD − PRESS) / SD

In this equation, SD is the sum of the squared deviations between the agonistic activities of the test set and the mean activity of the training molecules and PRESS is the sum of squared deviations between predicted and actual activity values for each molecule in the test set [13,24,25,26].

### 3.7. Models Validation-External Validation

The previous researches provided the fact that a high cross-validated correlation coefficient, *r*^2^_cv_, was the necessary condition for a robust predictive power but not a sufficient condition. The only way to estimate the true predictive ability of the model was external validation. A reliable 3D-QSAR model should have a robust predictive ability, if it is close to the ideal one. 3D-QSAR models were considered acceptable if they satisfy all of the set of criteria for evaluation of predictive ability of QSAR models [27,28,29,30]:

*r^2^_cv_*> 0.5, *r^2^* > 0.6, [(*r^2^ − r_0_^2^*)/*r^2^*] < 0.1, 0.85 ≤ k ≤ 1.15 and *r*^2^_m_ > 0.5

In this paper, the CoMFA and CoMSIA models were subjected to rigorous external validation process. We calculated the statistical parameters of the test set according to references [27,28,29,30].

## 4. Conclusions

We have employed 3D-QSAR to explore the structure-activity relationship of a series of 11*H*-dibenz[*b*,*e*]azepines and dibenz[*b*,*f*][1,4]oxazepines derivatives as potent TRPA1 agonists. CoMFA and CoMSIA analyses were used to build statistically significant models with good correlative and predictive capability for the activator of the human TRPA1 receptor. These models could be used to predict the potencies of related structures. Based on the excellent results of the external validation, the models established in the present study may be robust and reliable for predicting new derivatives. Moreover, the analysis of contours for the models has provided a clue for the design of new analogues with improved affinity and higher potency.
